# Lysosomal aggregation of iron nanoparticles guided by multistage transformation induces potent ferroptosis

**DOI:** 10.1016/j.mtbio.2026.103384

**Published:** 2026-06-22

**Authors:** Huan Liang, Runyu Hu, Qingqing Lu, Jun Zhang, Bingyan Lu, Jie Yang

**Affiliations:** Jiangsu Co-Innovation Center of Efficient Processing and Utilization of Forest Resources, College of Science, Nanjing Forestry University, Nanjing, 210037, PR China

**Keywords:** Ferroptosis, Fenton reaction, Lysosomal aggregation, Immunogenic cell death, Iron nanoparticles

## Abstract

Ferroptosis, a cell death form driven by lipid peroxidation accumulation via iron-dependent Fenton reaction, has attracted substantial attention in cancer therapy. This process is strictly dependent on iron ion concentration and environmental acidity. However, the relatively weak acidity in the tumor cytoplasm may significantly impair the Fenton catalytic activity of endocytosed iron-based nanoparticles. Inspired by the intrinsic acidic vesicular compartments of tumoral lysosomes, a multistage size-switching strategy is proposed to effectively target the optimal “battlefields” for iron-based materials. Through rational engineering, nanotransformers (NTF) integrated with collagenase (CLG) achieve targeted disassembly for deep tumor penetration and lysosomal aggregation to sustain Fenton catalytic activity. Enlarged iron depots in lysosomes prevent exocytosis, ensuring prolonged catalytic generation of lipid peroxidation species to initiate and amplify ferroptosis, ultimately inducing lysosomal membrane permeabilization and altered organelle functions. Further analysis reveals that iron nanoparticle lysosomal aggregation-mediated ferroptosis can effectively trigger immunogenic cell death and elicit robust antitumor immune responses. This work demonstrates the potential of well-designed multistage size-switchable nanotransformers in cancer treatment, representing a paradigm shift in advancing iron-based nanoparticle-mediated ferroptosis therapy.

## Highlights

• Trapping iron nanoparticles in acidic lysosomes to form iron depots sustains efficient Fenton catalytic activity.
• A multistage size-switching strategy is proposed to target the optimal “battlefields” for iron-based materials.
• Nanotransformers exert14-fold stronger lipid peroxidation induction than traditional cytosolic delivery strategies.
• The accumulation of lipid peroxidation species in lysosomes can initiate and amplify ferroptosis.

## Graphical abstract

Iron nanoparticles mediated ferroptosis is bottlenecked by limited iron release rate and poor catalytic efficiency in cytoplasm (pH 7.2-7.4). Herein, we reveal that trapping the iron nanoparticles in the acidic lysosomes (pH 4.5-6.0) to form iron depots constitutes a highly efficient strategy to address the aforementioned issues, achieving a 14-fold higher lipid peroxidation (LPO)-inducing capacity compared with traditional cytosolic delivery strategies.

**Figure:**
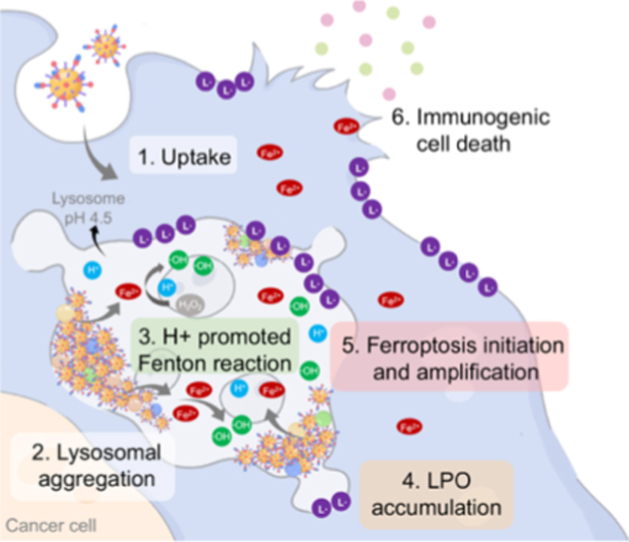

## 1 Introduction

Iron-based nanomaterials have garnered significant attention in the realm of cancer therapy due to their unique physicochemical properties, biocompatibility, and versatile functionalization [1,2]. Specifically, their released iron ions can drive the decomposition of hydrogen peroxide (H_2_O_2_) to generate highly reactive hydroxyl radicals (·OH), a chemistry process broadly recognized as the Fenton reaction [3]. Once internalized by cancer cells, iron-based nanoparticles can initiate and amplify radical chain reactions, promoting the formation of oxidized organic species and ultimately triggering cell death know as ferroptosis [4]. This procedure is strictly dependent on iron ion concentration and environmental acidity [5]. However, the relative weak acidity (pH 7.2-7.4) in tumor cytoplasm can significantly impair the Fenton catalytic activity of endocytosed iron-based nanoparticle [6,7]. This impact arises primarily from altered ion release behaviors and perturbed reaction microenvironments, ultimately weakening catalytic efficiency and resulting in suboptimal therapeutic outcome [8,9]. Thus, thoroughly accounting for the intracellular microenvironment and engineering adaptive nanomaterials, which can target optimal “battlefields” to regulate their catalytic activity is pivotal for achieving superior therapeutic efficacy.

Lysosomes, often termed as cellular degradative hubs, are defined by a dynamic system of acidic vesicular compartments, a key characteristic critical for degrading biological macromolecules to maintain cellular homeostasis [7,10]. Their internal pH typically ranges from 4.5 to 6.0, significantly lower than the neutral cytoplasm [11]. Notably, this acidic lumen aligns with the optimum pH range of Fenton reactions (pH 3.0-5.0), endowing it with the potential to maximize ·OH production [12]. Thus, rationally engineering a Fenton reaction system capable of selective accumulation in tumor lysosomes is anticipated to be an ideal strategy. However, the journey of nanomedicines from intravenous injection to targeted accumulation in tumor lysosomes constitutes a highly intricate cascade, with each step imposing distinct and often conflicting requirements on their physicochemical properties [[13], [14], [15]]. Balancing these competing demands, including stealth properties for circulation, small size for tissue penetration, receptor affinity for cellular uptake, and lysosomal aggregation, necessitates precise engineering design. Owing to aberrant tumor metabolism, excessive H^+^ and adenosine triphosphate (ATP) accumulate in the tumor microenvironment, resulting in a lower extracellular pH (around 6.5) and relatively high ATP concentrations ranging from 0.1 to 0.4 mM [16,17]. Additionally, tumor lysosomes exhibit a highly acidic, protein-rich environment containing over 60 types of enzymes [18]. Inspired by these nature features, we hypothesize that the uniquely distinct pathological microenvironment of tumor cells can be exploited as a selective trigger, effectively addressing the demands of the aforementioned multistage processes.

**Scheme 1:**
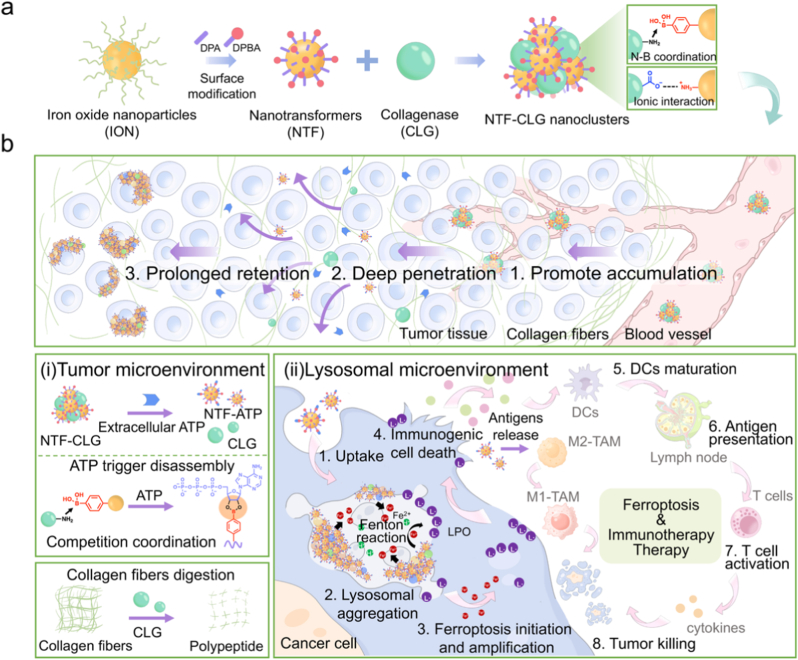
**Schematic illustration of the lysosomal aggregation of iron nanoparticles triggers robust ferroptosis.** (a) The fabrication of nanoclusters (NTF-CLG) consisted of nanotransformers (NTF) and collagenase (CLG) through a combination of nitrogen-boronate (N-B) coordination and ionic interactions. (b) Schematic illustration of NTF-CLG targeted aggregation in lysosomes to induce ferroptosis and immunogenic cell death (ICD) for cancer immunotherapy. During blood circulation, the formation of nanoclusters can prolong the circulation half-life and enhance tumor accumulation. Within the tumor microenvironment, extracellular adenosine triphosphate (ATP) initiates the disassembly of nanoclusters, leading to the release of NTF and CLG. Under the combined actions of size reduction and the CLG digestion of collagen fibers, NTF can achieve deeper penetration. Once endocytosed into cancer cells, the NTF are aggregated in lysosomes to induce Fenton reaction, and ultimately inducing ferroptosis and evoking ICD-associated antitumor immune.

To conduct a proof-of-concept trial, we demonstrated nanoclusters capable of switching sizes in the multistage pathological microenvironment of tumors, so as to effectively promote tumor permeation and selective lysosomal aggregation (Scheme 1). These rationally engineered nanoclusters integrate modified iron oxide nanoparticles (ION) and proteins. The ION have been extensively explored and were utilized as model Fenton reaction particles in our research. Initially, ION were decorated with mixture ligands of dopamine (DPA) and phenylboronic acid (PBA), and these modified nanoparticles were designated as nanotransformers (NTF). Subsequently, collagenase (CLG) was introduced and loaded onto NTF through multiple forces, thus forming nanoclusters (named NTF-CLG). The ribose structure in extracellular ATP has a stronger affinity for PBA [19], thereby disrupting the interaction between the protein and NTF, and effectively triggering the disassembly of NTF-CLG clusters. The released CLG acts as a scavenger to digest collagen fibers, promoting the intratumoral penetration of small-sized NTF particles and thus providing more opportunities for cellular uptake [20]. Subsequently, after entering tumor cells via endocytosis and being transported into lysosomes, the surface ligands of NTF interact with lysosomal proteins under acidic conditions. This interaction drives the further formation of relatively larger-sized iron depots, effectively preventing their elimination from lysosomes. After that, NTF can continuously catalyze the Fenton reaction in the acidic environment of lysosomes and initiate cellular ferroptosis. The oxidation of chemically reactive lipids within biological membranes leads to the accumulation of damaged phospholipids, which eventually causes lysosomal membrane permeabilization (LMP) along with altered organelle functions. Then, this alteration triggers the efflux of various chemical entities into the cellular plasma, which can induce further cellular defects and activate potent immunogenic cell death (ICD). Collectively, this well-designed gradient-driven multistage size-switchable strategy effectively seeks the optimal “battlefields” for the Fenton catalysis of iron-based materials, representing a paradigm shift in ferroptosis therapy and highlighting the potential to revolutionize highly specific and effective cancer immunotherapy.

## 2 Results and discussion

### 2.1 Size-switch behaviors of nanotransformers with different surface properties

**Fig. 1:**
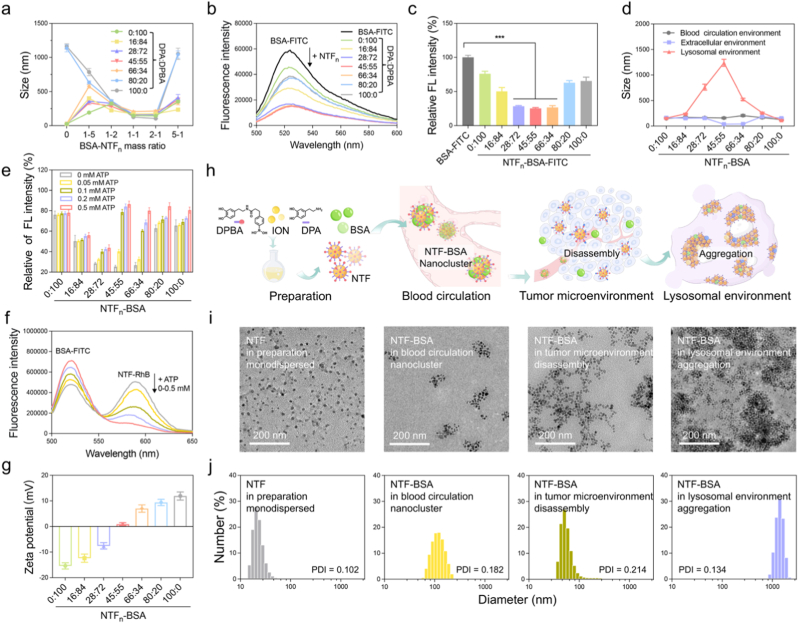
**Size-switch behaviors of nanotransformers upon different pathological environment.** (a) DLS analysis of NTF_n_-BSA with different formulations. (b) Fluorescence intensity changes of BSA-FITC with the introduction of NTF_n_. (c) Relative fluorescence intensity of BSA-FITC and NTF_n_-BSA-FITC nanoclusters with different ligand ratios. (d) Particle sizes of diverse NTF_n_-BSA nanoclusters under three distinct pathological microenvironments. (e) ATP-triggered BSA-FITC release from NTF_n_-BSA-FITC clusters (mean ± SD, n = 3). (f) FRET spectrum of NTF_n_-BSA-FITC with the increasing ATP treated, where FITC was the donor and RhB was the acceptor. (g) Zeta potential of NTF_n_-BSA in ALF solution (mean ± SD, n = 3). (h) Schematic illustration of pathological environment triggered multistage size-switch of nanotransformers. (i) TEM observation and (j) DLS measurement of nanotransformers with different pathological microenvironment.

The size switchable nanotransformers were synthesized using a ligand exchange method [21], which could form a nanoplatform consisting of iron oxide nanoparticles (ION) and several ligands. The ION was synthesized through a conventional thermal decomposition approach and served as model iron-based nanoparticles, which was equipped with Fenton reaction catalysis (detailed synthesis and characterization in Fig. S1–S3) [22,23]. The dopamine-phenylboronic acid (DPBA) was synthesized by amination reaction, furnishing a ligand platform for multi-stage size switching, whose boric structure could selectively form ester linkages with diol compounds [24]. The chemical structure of DPBA was validated by mass spectrometry and ^1^H NMR (Fig. S4 and S5). Taking into account the relationship between the surface potential and interparticle forces, the dopamine (DPA) was selected as a positive charged group. The molar ratios of DPA to DPBA are likely to determine the interactions among nanotransformers under different conditions. Therefore, a series of NTF samples (denoted as NTF_n_) with specific ligand ratios (*X*_DPA_: *X*_DPBA_ of 0:100, 16:84, 28:72, 45:55, 66:34, 80:20 and 100:0) was prepared and characterized to explore the interparticle forces. Subsequently, the bovine serum albumin (BSA) was employed as a model protein drug and loaded on NTF_n_ via the electrostatic binding and coordination interaction between phenylboronic acid and amino group of protein (denoted as NTF_n_-BSA) [25]. To achieve the optimal preparation, the NTF_n_ was blended with BSA at various weight ratio (w/w). As shown in Fig. 1a, upon the introduction of BSA, dynamic light scattering (DLS) measurements revealed a remarkable change in particle sizes, indicating the successful formation of nanoclusters. Specifically, when the BSA:NTF_n_ ratios were set at 1-1 and 1-2, the resulting diameters were more conducive to achieving an enhanced permeability and retention effect. Taking economic benefits into account, we selected the mass ratio of 1-1 as the final formulation for subsequent research.

To verify the successful loaded of BSA by NTF_n_, the aggregation caused quenching (ACQ) behavior of BSA-FITC was studied [26]. As depicted in Fig. 1b and c, upon the introduction of NTF_n_, the fluorescence intensity of FITC decreased. Notably, in the groups with *X*_DPA_: *X*_DPBA_ ratios of 28:72, 45:55, and 66:34, the ACQ effect of FITC was more prominent (approximately 25%). This result indicates that these three groups are more capable of forming optimal preparations for encapsulating BSA-FITC. Subsequently, we measured the particle sizes of diverse NTF_n_-BSA complexes under three distinct microenvironments: the blood circulation environment (pH 7.4, containing 10% fetal bovine serum, FBS), the extracellular environment (pH 6.5, with 0.1 mM adenosine triphosphate, ATP), and the lysosomal microenvironment (pH 4.5, containing 75% FBS) [[27], [28], [29]]. As show in Fig. 1d, in culture medium supplemented with 10% FBS, all types NTF_n_-BSA complexes remained stable and maintained a particle size of around 160 nm. As previously reported, the nanoparticles decorated with PBA could disassemble into small-sized particles due to the dynamic chemical reaction between the boric acid group on PBA and ATP. We conducted a further evaluation of the particle size alterations of NTF_n_-BSA in response to the increasing presence of ATP to simulate the tumor microenvironment (TME). As depicted in Fig. 1d, NTF_n_-BSA (45:55) and NTF_n_-BSA (66:34) groups demonstrated remarkable sensitivity to 0.1 mM ATP at pH 6.5. Upon the addition of ATP, their particle sizes decreased to approximately 40 nm. After confirming that the ATP could effectively trigger the selectively sensitive disintegrate of NTF_n_-BSA clusters, we were highly motivated to explore whether BSA could be released from NTF_n_ particles. The release behavior of BSA-FITC form diverse nanoclusters was studied by measuring the fluorescence recovery upon the addition of ATP. As shown in Fig. 1e, the groups with *X*_DPA_: *X*_DPBA_ ratios of 0:100, 16:84, and 28:72 displayed low sensitivity to the ATP, with only a small quantity of BSA-FITC being released. The groups with *X*_DPA_: *X*_DPBA_ ratio of 80:20 and 100:0 exhibited an unsatisfactory protein encapsulation effect. Notably, the NTF_n_-BSA (45:55) and NTF_n_-BSA (66:34) groups showed a dramatic response to ATP. After treatment with a relatively low concentration ATP of 0.1 mM (simulating the tumor microenvironment), approximately 60-80% of the fluorescence of BSA-FITC was recovered. In particular, the NTF_n_-BSA (45:55) not only achieved excellent protein encapsulation, but also could rapidly release the protein drug in response to ATP. For further verification, the disassembly of clusters triggered by ATP and the subsequent release of BSA were also demonstrated using Fluorescence Resonance Energy Transfer (FRET) technology [30]. Fluorescein isothiocyanate (FITC), which was labeled on BSA (BSA-FITC), and Rhodamine B (RhB), labeled on NTF (NTF-RhB), were employed as a FRET pair [31]. As presented in Fig. 1f, in the absence of ATP treatment, when excited at 488 nm, the emission peaks of both FITC and RhB could be detected, attributing to the energy transfer from FITC to RhB. However, after incubation with ATP, the emission peak of RhB decreased significantly, indicating that the BSA-FITC was released from the NTF particles. These changes of physicochemical properties in the presence of ATP in the simulated TME indicates the potential of these NTF_n_-BSA nanoclusters to respond effectively to the unique biochemical cues within the tumor site.

Upon incubation with 0.1 mM ATP, NTF_n_-BSA with DPA:DPBA ratios of 28:72, 45:55 and 66:34 was found to assemble into larger supraparticles with sizes ranging from approximately 500 to 1500 nm, when exposed to solutions mimicking the lysosomal environment (Fig. 1d). In contrast, nanoclusters modified with other ligand ratios showed only marginal growth. Additionally, in artificial lysosomal fluid (ALF, pH 4.5) supplemented with FBS, NTF_n_-BSA exhibited a lower surface potential as the proportion of DPBA increased, while a higher zeta potential was observed with an increase in the DPA ratio (Fig. 1g). When the molar ratio of DPA and DPBA was 45:55, NTF-BSA achieved an almost neutral charge (0.85 ± 0.57 mV). Previous reports have indicated that nanoparticles with mixed charge ligands are more prone to aggregation when reached electronegative neutrality through electrostatic interactions [32]. This aggregation behavior in the endosomal/lysosomal environment is of great significance for the retention and function of NTF_n_-BSA in tumor cells. Summing up all the scenarios, the NTF-BSA with *X*_DPA_:*X*_DPBA_ of 45:55 exhibited the most remarkable multistage size-switchable behavior in response to the tumor pathological microenvironment (Fig. 1h).

In addition, the NTF-BSA nanoclusters with *X*_DPA_:*X*_DPBA_ of 45:55 maintained favorable size and uniform dispersion within 48 h under both storage condition and serum-containing physiological environments, showing good stability during storage and blood circulation (Fig. S6). Consequently, it was selected as the bioactive nanocluster for subsequent research and was denoted as NTF-BSA. Moreover, Fourier-transform infrared spectroscopy (FTIR) and thermogravimetric analysis were employed to further validate the successful surface modification of the nanotransformers and the subsequent formation of NTF-BSA nanoclusters (Fig. S7 and S8). The morphology of the nanotransformers was observed, and DLS measurements were carried out in the preparation environment (pH 7.4), blood circulation environment (containing FBS, pH 7.4), extracellular environment (in the presence of ATP, pH 6.5), and lysosomal environment (in ALF, pH 4.5). As depicted in Fig. 1i, the synthesized NTF particles were highly dispersed and exhibited a uniform morphology, with a particle size of approximately 15 nm. Following incubation with BSA, the NTF-BSA nanoclusters were successfully obtained, with an average diameter of 100 nm. However, this assembly behavior can be disrupted by a weakly acidic environment (pH 6.5) with low-concentration ATP (0.1 mM), which precisely represents the pathological state of the tumor microenvironment [33]. We have noted that nanoparticles with smaller dimensions are more conducive to achieving deep-seated tumor penetration, strongly suggesting that NTF-BSA holds a promising application prospect in the controllable delivery of proteins within the tumor microenvironment. Moreover, the evolutionary aggregation into supra-clusters was further observed under conditions characteristic of lysosomes/endosomes, manifest as a low pH value and high protein concentration. The DLS results are in accordance with the above-mentioned analysis under different conditions (Fig. 1j). Intriguingly, our findings reveal that the multi-stage size switching of nanotransformers is spatiotemporally dependent. Notably, NTF-BSA alone did not aggregate under acidic and protein-rich conditions (Fig. S9). It is important to note that the further aggregation of nanoparticles within the artificial lysosomal environment is contingent upon their prior disassembly in the tumor microenvironment.

It could be concluded that the tumor pathological microenvironment can effectively trigger a step-by-step double transformation of NTF-BSA. This process enables the on-demand release of NTF in the extracellular environment, while simultaneously leading to its selective aggregation within the lysosomal space. Notably, the multistage size-switching property is likely to be advantageous for the deep penetration as well as subsequent aggregation induced retention in the tumor.

### 2.2 Tumoral lysosome-specific aggregation of nanotransformers

**Fig. 2:**
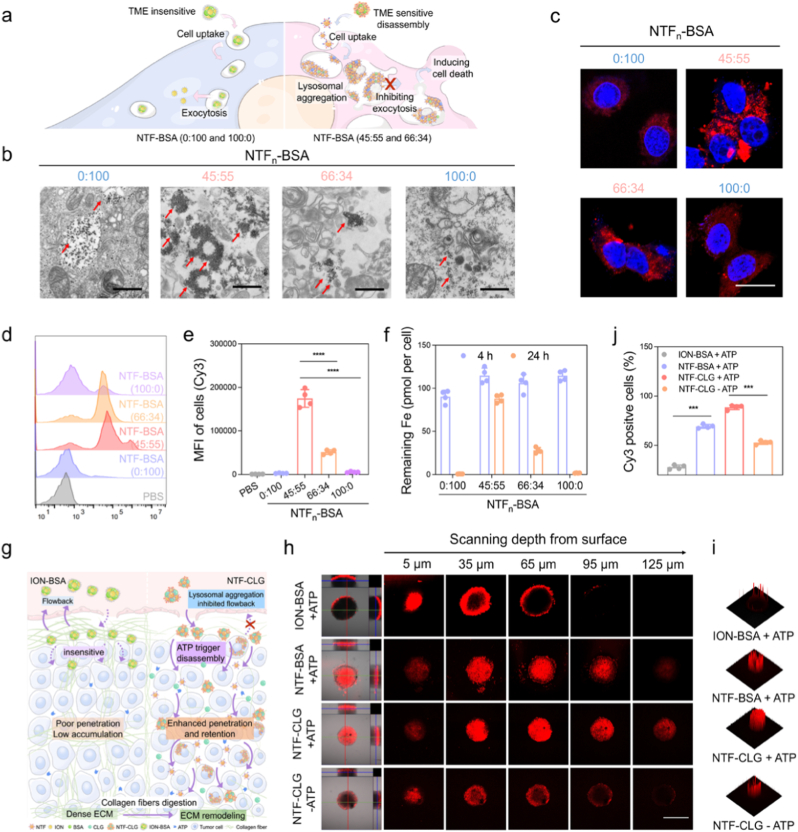
**Tumoral lysosome-specific aggregation of nanotransformers.** (a) Schematic of the lysosomal aggregation pathways for NTF_n_-BSA (45:55 and 66:34) (right) versus NTF_n_-BSA (0:100 and 100:0) (left) in cancer cells. (b) Representative TEM images of 4T1 cells treated by NTF_n_-BSA with different ligand ratios. The red arrows indicate the iron nanoparticles. Scale bars, 500 nm. (c) Confocal images of 4T1 cells after incubation with different formulations. NTF_n_ was labelled with Cy3, cell nuclei were counterstained with 4,6-diamidino 2-phenylindole (DAPI). Scale bar, 20 μm. (d) Flow cytometry analysis of 4T1 cells after incubation with nanoclusters. (e) Statistical data of flow cytometry for 4T1 cells after incubation with various NTF_n_-BSA. (f) Quantification of the Fe ions amounts remaining inside 4T1 cells following nanoclusters internalization. (g) Schematic illustration of the penetration of NTF in tumor. The penetration and retention of NTF are significantly increased, owing to the combined action of nanotransformers multistage size-switch and collagenase digestion of collagen fibers. (h) The CLSM images of 4T1 multicellular spheroids (MCSs) after incubating with Cy3 labeled nanoclusters with (+) or without (−) ATP supplement. 2.5D diagram of 95 μm depth sections of 4T1 MCSs after treatment. Scale bar, 500 μm. (i) Statistical data of flow cytometry for 4T1 MCSs cells after incubation with various nanoclusters. Data are presented as mean ± standard deviation (n = 4). (For interpretation of the references to colour in this figure legend, the reader is referred to the Web version of this article.)

The release of BSA and NTF from nanoclusters offers an opportunity for subsequent reactions on the cell membrane or within intracellular substructures. Specifically, NTF might be phagocytosed by tumor cells and selectively aggregate in the endosomal/lysosomal space (Fig. 2a). Take this hypothesis in mind, we first investigate the lysosomal aggregation behaviors of NTF-BSA with different ligands decorated ratios. As shown in bio-transmission electron microscopy (bio-TEM), the nanotransformers with *X*_DPA_: *X*_DPBA_ ratio of 45:55 exhibited a distinct aggregated distribution in the lysosome, which was consistent with the previous results. In contrast, the NTF_n_-BSA with an *X*_DPA_: *X*_DPBA_ ratio of 66:34 only showed a slight aggregation. The purely ligand-based nanoclusters (*X*_DPA_: *X*_DPBA_ ratios of 0:100 and 100:0) displayed a dispersed distribution and could be released from lysosomes into the cytoplasm or expelled from cells through exocytosis (Fig. 2b).

For the purpose of direct visualization, Cy3 labeled NTF was encapsulated within the clusters to track their uptake behavior at cellular level. As revealed by the confocal laser scanning microscope (CLSM) images, a much stronger fluorescence signal of Cy3 was detected in the cells treated with NTF-BSA (45:55) (Fig. 2c). Additionally, it could be observed that the red fluorescence of NTF-BSA with *X*_DPA_: *X*_DPBA_ ratios of 45:55 and 66:34 exhibited an aggregated distribution, indicating their second-stage transformation within the intracellular environment. The cellular retention of NTF was further analyzed by flow cytometry, and the results are presented in Fig. 2d and e. After 24 h treatment, the NTF_n_-BSA with pure ligands demonstrated negligible retention in 4T1 cells, which might be attributed to the rapid efflux of tumor cells as a self-protection mechanism. Notably, a significantly increased Cy3 signal was observed following treatment with NTF_n_-BSA (45:55). This enhanced cellular uptake and retention behavior of NTF-BSA with a specific ligand ratio provide strong evidence for its effectiveness in tumor targeting and intracellular delivery, which is crucial for improving the therapeutic efficacy of nanoparticle-based cancer therapies.

The final amount of iron retained in the cells was determined by inductively coupled plasma-optical emission spectrometry (ICP-OES) to reflect the aggregation of nanoclusters. As presented in Fig. 2f, after 4 h incubation, there was little disparity in the cellular uptake of each nanocluster. Nevertheless, when the incubation time was extended to 24 h, almost all iron particles were eliminated from the cells in the groups of NTF_n_-BSA decorated with pure ligands. Even in the NTF-BSA (66:34) group, approximately 75% of the internalized NTF_n_ were secreted from the cells. A quite different result was observed in the cells treated with NTF-BSA (45:55). The exocytosis efficiency in NTF-BSA (45:55) group was significantly reduced, and around 77% of the NTF could be retained in the tumor cells within the same time period. This phenomenon clearly demonstrates that an appropriate ligand modification ratio enables the selective retention of nanoparticles in cells due to their lysosomal aggregation.

As is well known, the solid tumors possess a dense extracellular matrix (ECMs), with collagen fibers being particularly prominent [34]. These collagen fibers give rise to increased solid stress, which acts as a significant barrier, impeding the penetration of nanoparticles [35]. Therefore, merely controlling the particle size is not the optimal approach to overcome the challenge of deep tumor drug delivery. In this study, we introduce the collagenase (CLG) as a functional protein drug, which can digest the collagen fibers (Fig. 2g). Notably, BSA and CLG possess similar molecular weights and isoelectric points, suggesting that the encapsulation behavior of NTF with CLG may be similar to that with BSA. Actually, the NTF-CLG exhibited comparable size variations to NTF-BSA, verifying the generalizability of the established mechanism (Fig. S10). On the basis of the above characterizations and validations, we further constructed 4T1 multicellular spheroids (MCSs) to verify whether CLG loaded nanotransformers (NTF-GLC) could promote the penetration of particles in tumors trough dual effect of collagen degradation and size switching [36]. Cy3 labeled nanoclusters were incubated with MCSs under specific conditions. The penetration behavior of particles was observed by CLSM through Z-stack scanning. As shown in Fig. 2h, the NTF with pure dopamine modification (denoted as ION-BSA) displayed a limited penetration capability, owing to the fixed particle diameter. After incubation with ATP, the permeability of NTF-BSA increased significantly to approximately 105 μm, indicating that the sensitive disassembly induced release of NTF small particles could effectively promote tumor penetration. Notably, NTF-CLG with CLG loading displayed excellent penetration ability upon incubation with ATP. Its red fluorescence was evenly distributed even at a scanning depth of 125 μm. On the contrary, in the NTF-CLG treated group without ATP, only faint red fluorescence was detectable at the edge of the MCSs in the 65 μm section. The fluorescence intensity and distribution within the spheroids were also analyzed using a 2.5 D diagram at the 95 μm depth section (Fig. 2i). Simultaneously, the treated MCSs were collected and further analyzed by flow cytometry to evaluate the cell uptake behavior of the nanotransformers under the same conditions. As depicted in Fig. 2j and Fig. S11, the number of positive cells in the ION-BSA group with ATP incubation was extremely low, accounting for only 29.1%. While, the positive rate surged to a staggering 71.8% in the NTF-BSA + ATP group, mainly due to the disintegration of the nanotransformers into small particles within the simulated tumor microenvironment. For the CLG-loaded nanoclusters, under the simulated TME culture conditions (pH 6.5, 0.1 mM ATP), the positive cells in the NTF-CLG treatment group reached 89.8%, which was attributed to the digestion of collagen fibers. Under ATP-free conditions, the positive cell rate plummeted to 54.9%, which reflected the ATP-dependent targeting and collagenolytic activation of NTF-CLG nanoclusters. Different from the previous qualitative representative images, these quantitative results provide intuitive and numerical evidence, further confirming that the combined effect of ATP-induced size transition and CLG-based collagen degradation achieves an obvious synergistic enhancement in tumor deep penetration.

### 2.3 Nanotransformers aggregation induced ferroptosis

**Fig. 3:**
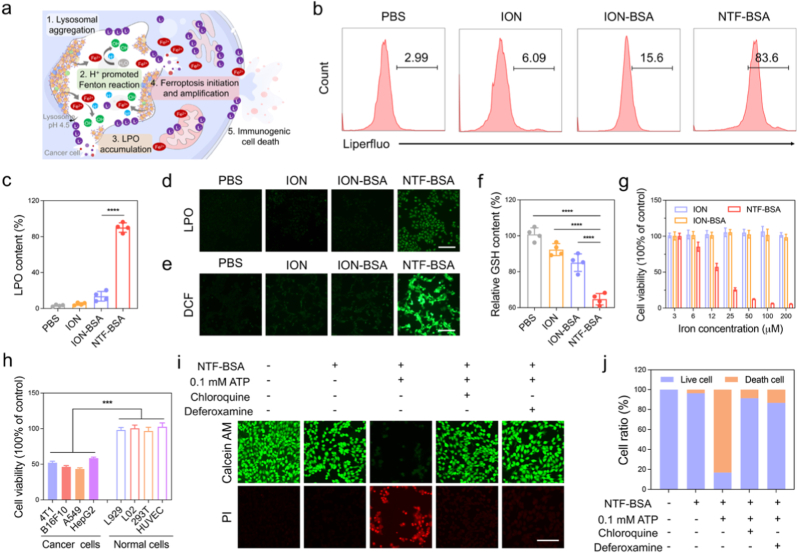
**Lysosome-specific aggregation of nanotransformers induces cell death.** (a) Schematic illustration of the lysosomal aggregation of NTF mediated iron overload to promote Fenton reaction, and ultimately initiate and amplify the ferroptosis for tumor cell killing. Purple circles represent lipid radicals. (b, c) LPO content estimated by flow cytometric quantification. (d) LPO levels observed by Liperfluo probe. Scale bar, 100 μm. (e) The ROS production in 4T1 cells subjected to various treatments using DCFH-DA as a probe. Scale bar, 100 μm. (f) Relative GSH content in 4T1 cells after different treatments. (g) Cytotoxicity for 4T1 cancer cells co-treated with various formulations for 24 h. (h) Cell viabilities of various cancer cells and normal cells after incubation with NTF-BSA at mimicking tumor microenvironment solution. (i) Calcein AM/PI staining of 4T1 cells after different treatments. Calcein-AM (green, live cells) and PI (red, dead cells). Scale bar, 100 μm. (j) Statistical analysis of (i). (n = 4, mean ± SD). ***P < 0.001, ****P < 0.0001, ns > 0.05. (For interpretation of the references to colour in this figure legend, the reader is referred to the Web version of this article.)

The selectively lysosomal aggregation and its subsequent transformation to coalesce into large clusters may cause iron overloaded and oxidation of lysosomal membrane lipids, which further aggravates lysosomal swelling, rupture and ultimately LMP (Fig. 3a). Therefore, typical markers were utilized to investigate the NTF induced ferroptosis. The fluorescence probe Liperfluo was used to evaluate the production of intracellular lipid peroxidation (LPO) [37]. As shown in Fig. 3b and c, The NTF-BSA induced an extensive increase of LPO accumulation in 83.6% 4T1 cells. In consistent, the much brighter green fluorescence signals were observed in NTF-BSA treated cells (Fig. 3d). The fluorescence intensity of LPO probe only moderately increase in the ION and ION-BSA groups. No obvious green fluorescence signal was observed in the groups treated with fixed-size nanoparticles (Fig. 3e). However, the cells treated with NTF-BSA exhibited markedly increase fluorescence signal, indicating the lysosomal aggregation of iron particles was necessary for reactive oxygen species (ROS) generation. As a powerful intracellular antioxidant, the glutathione (GSH) can protect cells from ferroptosis by inhibiting ROS, especially the cytotoxic LPO [38]. Therefore, the increase of GSH depletion can lead to the accumulation of LPO, and indirectly reflecting the ferroptotic Fenton reaction degree. As expected, the GSH level was significantly decreased in the NTF-BSA group relative to that in PBS treated group (Fig. 3f). While, the ION and ION-BSA treated cells only exhibited a faint level of GSH consumption, due to the limited accumulation of iron particles.

Subsequently, we aimed to investigate the intracellular tumor cell killing effect of NTF-BSA. As shown in Fig. 3g, the 4T1 cells exposed to NTF-BSA exhibited a dose dependent killing effect. In contrast, the cytotoxicity effect of ION and ION-BSA groups was marginal. Significantly, we discovered that NTF-BSA displayed selective cytotoxicity towards tumor cells. In total, we test 4 cancerous and 4 normal cell lines, and the results are presented in Fig. 3h. The half maximum inhibition concentration (IC_50_) of NTF-BSA in acidic TME (pH 6.5, 0.1 mM ATP) was approximately 14.8 μM for 4T1 cells, and this effect was also observed in other cancer cell types. Conversely, no significant cytotoxicity was detected in normal cells under physiological conditions. This result can be attributed to the limited aggregation of NTF in normal cells, which is due to their acid and energy metabolism homeostasis [39]. Therefore, the pathological microenvironment driven nanotransformer based ferroptosis depots cause minimal harm to normal cells. Tumor cell killing effect of nanoparticles was validated by propidium iodide (PI) staining [40]. As shown in Fig. 3i and j, intense red fluorescence was observed in the cells after treatment with NTF-BSA in an acidic TME mimicking culture medium. However, chloroquine, an inhibitor of endosomal acidification [41], could reverse the tumor killing effect of NTF-BSA, indicating the cell killing effect of NTF-BSA is strictly dependent on lysosomal acidity, which is consistent with our previous hypotheses. While, the deferoxamine (DFO) also impaired the tumor cell killing activity of NTF-BSA, due to the chelation of iron ions [42]. These findings collectively demonstrated that lysosomal iron aggregation is a crucial factor for NTF-BSA initiated ferroptotic cell death.

### 2.4 Evaluation of nanotransformer induced immune regulation effects in vitro

**Fig. 4:**
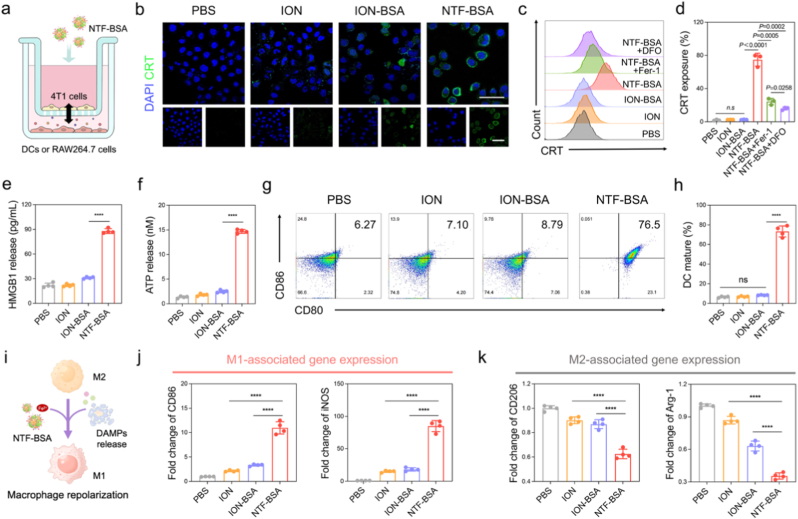
**Evaluation of nanotransformer induced immune regulation effects.** (a) Schematic illustration of the experiment design of DCs maturation or macrophage re-education in a transwell chamber. (b) CLSM images of CRT (green) exposure on the surface of 4T1 cells after different treatments. The cell nucleus was stained with DAPI (blue). Scale bar, 50 μm. (c) Flow cytometric analysis and (d) corresponding quantitative statistics of CRT exposure in 4T1 cells upon different treatments. (n = 3, mean ± SD). (e) HMGB1 released from 4T1 cells detected by enzyme-linked immunosorbent assay (ELISA) kit. (n = 4, mean ± SD). (f) ATP secretion detected by enhanced ATP assay kit. (n = 4, mean ± SD). (g, h) Representative flow cytometric plots and their quantitative analysis of DCs maturation after different treatment. (n = 4, mean ± SD). (i) Schematic illustration of NTF-BSA mediated macrophage repolarization. (j, k) Quantitative analysis of the M1-related makers (CD86 and iNOS) and M2-ralated genes (CD206 and Arg-1) in RAW264.7 cells subjected to different treatments by polymerase chain reaction. (n = 4, mean ± SD). ***P < 0.001, ****P < 0.0001, ns > 0.05. (For interpretation of the references to colour in this figure legend, the reader is referred to the Web version of this article.)

The ferroptosis has been highlighted as a potent inducer of immunogenic cell death (ICD) to further elevate tumor inmmunogenicity [38]. The ferroptotic cells underwent ICD to release danger-associated molecular patterns (DMAPs) as costimulatory signals to transform tumors from immune deserted phenotype into an immune inflamed state [43]. Therefore, the key DMAPs were evaluated in different formulations treated 4T1 cells through a transwell system (Fig. 4a). As shown in Fig. 4b, the cells treated with NTF-BSA revealed significantly elevated calreticulin (CRT) exposure. While, the ION and ION-BSA did not posse the capacity to upregulate CRT level. To verify the essential role of ferroptosis in triggering tumor ICD, we further performed flow cytometry to detect CRT exposure in 4T1 cells upon NTF-BSA treatment with or without Ferrostatin-1 (Fer-1) and DFO intervention, the well-established iron chelator and classical ferroptosis inhibitors. The results revealed that Fer-1 and DFO could remarkably reduce the surface translocation of CRT induced by NTF-BSA, confirming that ferroptosis is critically required for the ICD response (Fig. 4c and d). While, consistent with result of CRT exposure, the translocation of high-mobility group protein B1 (HMGB1) from the nucleus into the cytoplasm was remarkably increased in the NTF-BSA treated cells (Fig. 4e). Simultaneously, significantly elevated of ATP concentration in extracellular milieu was detected in 4T1 cells treated with NTF-BSA compared with other groups (Fig. 4f). All results collectively validated that the lysosomal aggregation of NTF particles could robustly initiate ferroptosis-based ICD, underscoring their extraordinary potential to enhance antitumor immune responses.

The tumor associated antigens (TAA) release and DMAPs exposure during ICD could facilitate dendritic cells (DCs) maturation and trigger adaptive immunity [44]. Therefore, the immature DCs were cocultured with 4T1 cells and subjected to various treatments to evaluate their maturation rate. Consistent with above results, the expression of costimulatory molecules (CD80 and CD86) was measured by flow cytometry. The DCs cocultured with NTF-BSA treated tumor cells exhibited much higher maturation rate (76.5%) than that of PBS group (6.27%) (Fig. 4g and h). Whereas, the proportion of mature DCs in ION and ION-BSA interacted groups only displayed negligible increase. In addition, the potential cytotoxicity of NTF-BSA towards DCs was evaluated to emphasis the safety of pathological microenvironment responsive multi-stage size switch strategy in nanotransformers based lysosomal aggregation. Fortunately, the NTF-BSA did not display significant cytotoxicity to DCs in the MTT assay (Fig. S12). Iron-based nanoparticles and the iron induced cancer cell death can create an autocrine feedback loop that efficiently mediate macrophage responses [45]. Therefore, regulation of tumor associated macrophage (TAM) from immunosuppressive M2 to pro-inflammatory M1 is a promising therapeutic strategy (Fig. 4i). Herein, the macrophage re-education was assessed by detecting the expression of key phenotype related markers by quantitative real-time polymerase chain reaction (RT-PCR). As shown in Fig. 4j and k, the RAW264.7 cells interacted with 4T1 cells that treated with NTF-BSA displayed remarkably upregulated M1-related makers (CD86 and iNOS) and decreased M2-ralated genes (CD206 and Arg-1). Of note, all iron-based formulations demonstrated similar ability to polarize the macrophages in case of directly exposure (Figs. S13 and S14). Collectively revealed that the iron-based directly regulation and lysosomal aggregation initiated ferroptotic cell death synergistically promoted the repolarization of tumor associated macrophage differentiation into antitumor phenotypes, which may potentiate TAM-modulating cancer immunotherapy.

### 2.5 Aggregation induced immunogenic cell death to enhance antitumor efficacy

**Fig. 5:**
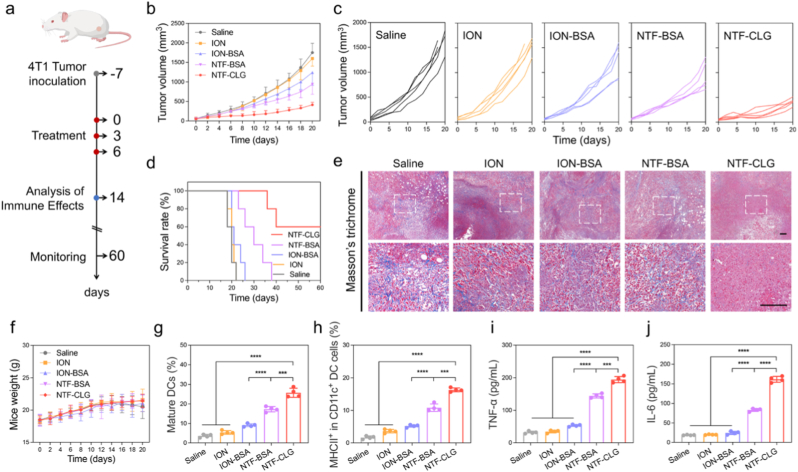
**Evaluation of the antitumor effects of nanotransformers in vivo.** (a) Scheme illustration of tumor therapy experiment design. (b) Average tumor growth curves and (c) individual tumor growth curves of 4T1 breast cancer after different formulations treatment. (n = 5, mean ± SD). (d) Survival percentages of tumor bearing mice after treatment in 2 months observation period. (e) After intravenous administration of different treatments to 4T1 tumor-bearing mice, tumors were collected and stained with Masson's trichrome. Scale bar, 200 μm. (f) Mice body weight after treatments. (n = 5, mean ± SD). (f) Flow cytometric analysis of mature DCs in lymph nodes following various treatments. (n = 4, mean ± SD). (h) Quantification of MHC II^+^ DCs populations by flow cytometry under different interventions. (n = 4, mean ± SD). (i) Serum TNF-α and (j) IL-6 levels in mice after treatment. (n = 4, mean ± SD). ****P < 0.0001, ***P < 0.001, **P < 0.01.

Inspired by these promising in vitro results, we proceeded to study the performance of nanotransformers in 4T1 tumor bearing BALB/c mice, as illustrated in Fig. 5a. For a more comprehensive comparison, the ION and ION-BSA with fixed particle diameter were employed as control group. The tumor volume was measured every two days, and the curves are presented in Fig. 5b and c. ION and ION-BSA demonstrated a modest tumor suppressing activity, which might be ascribed to its inherent chemodynamic therapeutic activity, as previously reported [46]. The NTF-BSA group significantly inhibited tumor growth, with an inhibition rate of approximately 24.5%. As anticipated, on day 20, the tumor volumes in mice treated with NTF-CLG were only approximately 33.1% of those in the saline treated group. These results highlight the crucial role of multistage size-switching nanotransformers mediated lysosomal aggregation and the subsequently initiation of ferroptosis induced tumor cell killing effect. Consistent with these findings, the mice treated with NTF-CLG exhibited a substantially prolonged survival time, with a final survival rate of 60% after a 2 months observation period (Fig. 5d). Subsequently, we evaluated the collagen-depletion efficacy of CLG-loaded nanoclusters in vivo (Fig. 5e). Masson's trichrome staining of tumor tissues from NTF-CLG treated mice revealed a remarkable reduction in collagen fibers, directly validating the efficient extracellular matrix (ECM) degradation mediated by collagenase in vivo. Importantly, no obvious body weight fluctuations were observed throughout the entire treatment period, supporting the favorable biosafety of the nanoclusters (Fig. 5f).

According to recent research, ferroptosis associated cell death in tumor cells typically exhibits high immunogenicity [47]. Therefore, the changes in the immune landscape within lymph nodes and tumors were determined to investigate whether NTF-CLG mediated lysosomal aggregation and the formation of iron depots can trigger immunogenic cell death. The limited DCs maturity is a key performance of low immunogenicity of tumors, which suppressing the cancer immunotherapy. Given the impacts of nanotransformers on the activation of ICD, DCs maturation and activity restoration for antigen presentation was initially studied. As shown in Fig. 5g and Fig. S15, the matured DCs (represented by CD80^+^CD86^+^ in CD11c^+^ cells) in nanotransformers treated groups were significantly increased versus the saine group (3.24%). As expect, the mice treated with NTF-CLG exhibited the highest proportion of DCs maturity of about 28.8%, indicating the synergistic effect of NTF-based lysosomal-selected aggregation and CLG mediated collagen degradation to stimulate the immune system. Besides, the antigen presentation ability of DCs was further evaluated by MHC II expression (MHCII^+^CD11c^+^). As determined by FCM in Fig. 5h and Fig. S16, the MHC II expression in NTF-CLG treated mice was increased to 16.3%, surpassing the values observed in the NTF-BSA (10.7%) group, demonstrating a remarkable promoted capability in antigen presentation as described above. Additionally, the treatment with NTF-CLG significantly elevated the levels of tumor necrosis factor-α (TNF-α) and interleukin (IL-6) secreted by immune cells (Fig. 5i and j), indicating robust activation of immune responses. Collectively, these results highlight the therapeutic potential of NTF-CLG to trigger ferroptosis and modulate immune system, offering a promising avenue for the development of effective and selective anticancer therapies.

**Fig. 6:**
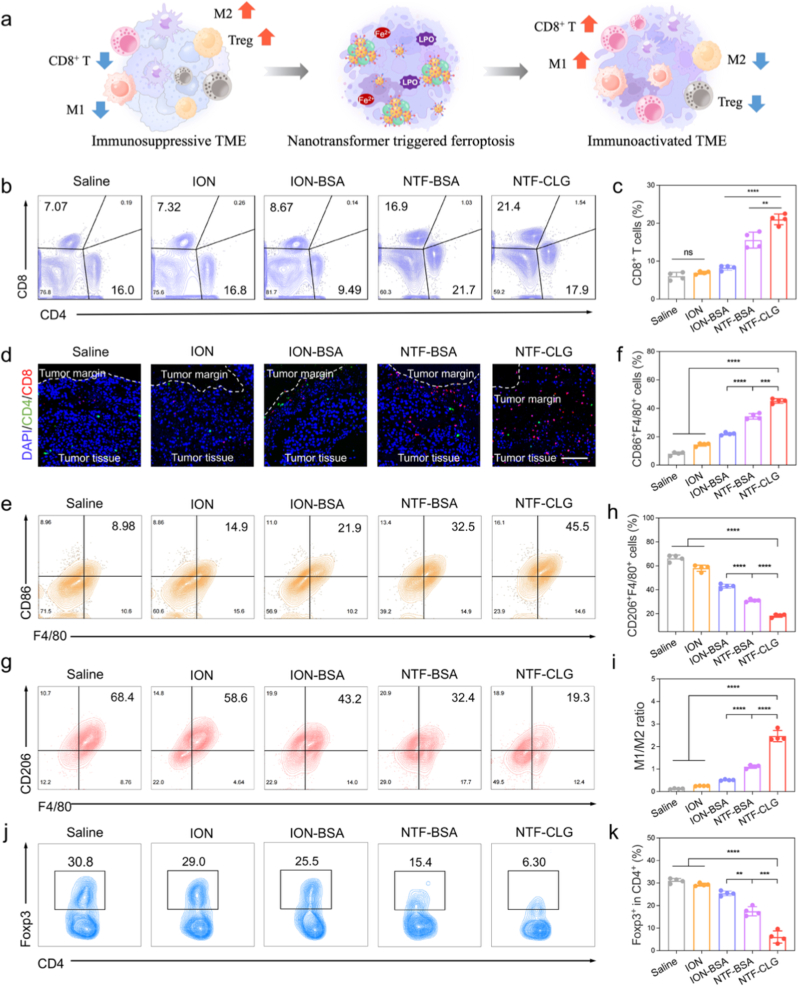
**Evaluation of the antitumor immune responses induced by lysosomal aggregation of iron nanoparticles.** (a) Scheme illustration of immune regulatory effects induced by NTF-CLG nanoclusters. (b) Representative flow cytometric analysis and (c) corresponding quantification results of cytotoxicity T Lymph cells infiltration within the tumors. (n = 4, mean ± SD). (d) Immunofluorescence staining of CD8^+^ (red) and CD4^+^ (green) T lymph cells in tumor tissues after different treatments. Nuclei were stained with DAPI (blue). Scale bars, 100 μm. Flow cytometry quantitative analysis of (e, f) M1-TAM and (g, h) M2-TAM in tumor tissues after different treatments. (n = 4, mean ± SD). (i) The ratios of M1/M2 macrophages in tumors of 4T1 tumor-bearing mice following various treatments. (j) Representative flow cytometric analysis and (k) corresponding quantification results of Tregs infiltration within the tumors. (n = 4, mean ± SD). ****P < 0.0001, ***P < 0.001, **P < 0.01, ns > 0.05. (For interpretation of the references to colour in this figure legend, the reader is referred to the Web version of this article.)

It is important to acknowledge that the immunosuppressive tumor microenvironment tends to hinder the functionality of immune cells, with a particular propensity to compromise the cytotoxic capabilities of effector T cells. This represents a significant barrier to the further activation of antitumor immune responses, ultimately contributing to the failure of therapeutic interventions (Fig. 6a). Therefore, intratumorally infiltration and expansion of cytotoxicity T cells were further evaluated. As shown in Fig. 6b and c, following NTF-CLG treatment, the proportion of CD8^+^ T cells increased to 21.4%, which was significantly higher than that in the NTF-BSA treated mice (16.9%) and the saline treated group (7.07%). The enhanced infiltration and activation of CD8^+^ T cells in tumor tissues were further confirmed by immunofluorescence staining (Fig. 6d). The fluorescence signal of CD8^+^ T cells in the tumor section treated with NTF-CLG was much brighter than that in other groups. All results demonstrated that the NTF-CLG could effectively facilitated the cell activation and infiltration of CD8^+^ T in tumor.

However, the immunosuppressive TEM promoted the TAMs polarized to anti-inflammatory M2 phenotype and impaired the activity of CD8^+^ T cells [48]. Therefore, re-education TAMs polarization from the M2 phenotype to the pro-inflammatory M1 one represents a promising strategy to reverse the immunosuppressive status, restore the activity of cytotoxicity T cells and enhance antitumor immunity. Inspired by the unique physicochemical properties of iron-based nanoparticles for promoting the macrophages polarization toward the M1 phenotype by affecting intracellular iron ion concentration, altering cell metabolic types, and activating inflammatory signaling pathways. Therefore, the phenotype of macrophages in tumor tissues after various treatments were measured by flow cytometry. As shown in Fig. 6e and f, the nanotransformers significantly remarkably elevating the recruitment of M1-type TAM (CD86^+^F4/80^+^ gated in CD11b^+^ cells) in tumors. Particularly, the M1-type macrophages in NTF-CLG group (45.5%) were around 5-fold versus to that in the saline group (8.98%). While, the proportion of M2-type macrophages (CD206^+^F4/80^+^ gated in CD11b^+^ cells) reduced from 68.4% in the saline group to 32.4% in NTF-BSA group, and further decreased to 19.3% after NTF-CLG treatment (Fig. 6g and h). This TAMs re-education shifted from M2 to M1 was most pronounced in the NTF-CLG group versus to other groups (Fig. 6i). Furthermore, a striking reduction in the recruitment of immunosuppressive regulatory T cells (Tregs) was noted. As illustrated in Fig. 6j and k, the proportion of intratumoral Tregs plummeted from 30.8% in the saline treated group to 6.30% in the NTF-CLG administered group. This phenomenon collectively supported the synergistic immune activation potential of NTF and CLG.

To systematically evaluate the in vivo biosafety and biocompatibility of NTF-CLG, comprehensive safety assessments were performed in healthy mice. The animals were randomly divided into groups and administered different formulations via three consecutive injections to assess potential systemic toxicity. As presented in Fig. S17, all major hematological parameters, including red blood cells (RBC), white blood cells (WBC), hemoglobin (HGB), platelets (PLT), monocytes (Mon), lymphocytes (Lym), and neutrophils (Neu), remained within normal physiological ranges. Similarly, liver function markers (aspartate aminotransferase, AST; alanine aminotransferase, ALT) and renal function indicators (blood urea nitrogen, BUN; creatinine, CREA; uric acid, UA) showed no obvious abnormalities. Furthermore, the hematoxylin-eosin (H&E) staining of major organs (heart, liver, spleen, lung and kidney) was performed to examine pathological damage, inflammation or tissue abnormality. As shown in Fig. S18, no obvious organ injury or inflammatory lesions were observed in the NTF-CLG group compared with the control groups. These results collectively demonstrated that NTF-CLG caused negligible systemic toxicity, further supporting the promising potential of such nanotransformers for further biomedical applications.

## 3 Conclusions

This study presents a gradient-driven, multistage size-switchable nanocluster system (NTF-CLG) that strategically addresses the key challenges of iron-based nanomaterial delivery and Fenton catalysis in cancer therapy. By leveraging the unique pathological microenvironment of tumors, the system achieves targeted disassembly for deep tumor penetration and lysosomal aggregation to maintain sustained Fenton activity. The formation of iron depots in lysosomes prevents excretion, ensuring prolonged catalytic generation of ·OH, which triggers ferroptosis through lipid oxidation and lysosomal membrane permeabilization (LMP), ultimately activating immunogenic cell death (ICD). Both in vitro and in vivo results demonstrated the robust antitumor efficacy of nanotransformers, which remarkably promoted DCs maturation and TAMs re-education, as well as increased the infiltration of cytotoxicity T cells infiltration. This design exemplifies how engineering nanomaterials to adapt dynamically to intracellular and extracellular cues can overcome the conflicting demands of circulation, penetration, uptake, and retention. By homing in on lysosomes as optimal “battlefields” for Fenton reactions, the strategy maximizes therapeutic efficacy while minimizing side effects, offering a paradigm for precision ferroptosis-based cancer therapy.

Despite the promising therapeutic outcomes, several limitations of the current NTF-CLG system warrant consideration. First, the in vivo evaluation was primarily conducted in immunocompetent murine models with relatively homogeneous tumor burdens, which may not fully recapitulate the complexity of human solid tumors. Additionally, the ICD-induced antitumor immunity, although enhanced, may still be constrained by the immunosuppressive TME, such as the presence of Tregs and myeloid-derived suppressor cells (MDSCs), which could limit the durability of antitumor responses. While, the fabrication process of NTF-CLG involves multistep modification, which may increase production complexity and cost, posing challenges for potential clinical translation. To address these limitations and expand the therapeutic potential, future efforts can be directed toward multiple complementary directions. Optimizing the nanocluster's surface properties, such as modifying with tumor-specific ligands to enhance targeting specificity, thereby reducing off-target accumulation and improving deep penetration in highly heterogeneous tumors. Integrating NTF-CLG with immune checkpoint inhibitors (e.g., *anti*-PD-1/PD-L1 antibodies) or small-molecule immunomodulators offers a synergistic approach to reverse TME immunosuppression, further boosting ICD-mediated antitumor immunity and preventing tumor recurrence. Besides, developing a simplified and scalable fabrication strategy is also critical to improving production efficiency and reducing costs, which will facilitate the smooth transition from preclinical research to clinical development. Additionally, we can explore combinations of NTF-CLG with other therapeutic modalities, such as photodynamic therapy or radiotherapy, to achieve multimodal synergistic effects that may overcome ferroptosis resistance in certain tumor subtypes. Collectively, these advancements are expected to refine the gradient-driven nanocluster platform and promote its broader application in precision cancer therapy.

## CRediT authorship contribution statement

**Huan Liang:** Conceptualization, Data curation, Funding acquisition, Project administration, Validation, Visualization, Writing – original draft, Writing – review & editing. **Runyu Hu:** Data curation, Formal analysis, Investigation. **Qingqing Lu:** Data curation, Methodology. **Jun Zhang:** Data curation, Investigation. **Bingyan Lu:** Data curation, Formal analysis. **Jie Yang:** Supervision, Validation, Visualization, Writing – review & editing.

## Declaration of competing interest

The authors declare that they have no known competing financial interests or personal relationships that could have appeared to influence the work reported in this paper.

## Data Availability

Date will be made available on request.
