# Supplementary material for: Lysosomal aggregation of iron nanoparticles guided by multistage transformation induces potent ferroptosis
Source: Mater Today Bio. 2026 Jun 22;39:103384. doi: 10.1016/j.mtbio.2026.103384 (PMC13320257; doi:10.1016/j.mtbio.2026.103384)
Supplement: Multimedia component 1 [file mmc1.docx]

Supporting Information

**Lysosomal Aggregation of Iron Nanoparticles Guided by Multistage Transformation Induces Potent Ferroptosis**

Huan Liang*, Runyu Hu, Qingqing Lu, Jun Zhang, Bingyan Lu, Jie Yang*

Jiangsu Co-Innovation Center of Efficient Processing and Utilization of Forest Resources, College of Science, Nanjing Forestry University, Nanjing 210037, P. R. China

Email: [hliang1005@njfu.edu.cn](mailto:hliang1005@njfu.edu.cn); [jieyang@njfu.edu.cn](mailto:jieyang@njfu.edu.cn)

Experimental Section

**Materials:** Iron (Ⅲ) acetylacetonate, Benzyl ether, Oleylamine were obtained from Sigma Aldrich. Dopamine hydrochloride (DPA) and 4-Carboxylphenylboronic acid pinacol ester (PBA-PE) acid were obtained from Energy Chemical. Adenosine triphosphate (ATP), N,N-Diisopropylethylamine (DIPEA), 2-(7-Azabenzotriazol-1-yl)-N,N,N',N'-Tetramethyluronium Hexafluorophosphate (HATU) and deferoxamine (DFO) were purchased from Aladdin chemical regent company. Protein of collagenase (CLG) and antibodies used in this work were purchased from Cell Signalling Technology or Biolegend. Fluorescin isothiocyanate (FITC), rhodamine B (RhB), Cy3 were acquired from RuixiBio Tech. Bovine serum albumin (BSA), 4',6-diamidino-2-phenylindole (DAPI), 2’, 7’-Dichlorodihydrofluorescein diacetate (DCFH-DA) assay kit, DMEM medium and PBS were from Solarbio. Calcein AM / prodium iodide (PI) cell apoptosis assay kit, BCA protein assay kit, reduced glutathione (GSH) assay kit, liperfluo probe and methyl thiazolyl tetrazolium (MTT) were obtained from KeyGen Biotech. Mouse TNF-α and IL-6 ELISA kits were purchased from Wuhan Boster Biological Technology Co. Ltd.

**Synthesis of ligand:** The ligand was synthesized by the amination reaction. Briefly, PBA-PE (248 mg, 1 mmol) was dispersed in 10 mL dimethyl formamide (DMF) and HATU (456 mg, 1.2 mmol), DIPEA (155 mg, 1.2 mmol) were added as the catalyst. Subsequently, the DPA (284.5 mg, 1.5 mmol) was added to above mixture. The mixture was maintained under nitrogen protection with gentle stirring for 6 h. Then, the organic phase was concentrated and dried by rotary evaporation. The crude products were further purified with column chromatograph using DCM:methanol = 10:1 (v/v) to receive pure white powder (DPBA-PE). The chemical structure was characterized by ^1^H-NMR and mass spectrograph.

**Preparation of coated iron oxide nanoparticles (NTF):** The magnetic iron oxide nanoparticles (ION) were synthesized by high temperature pyrolysis method. Briefly, iron (Ⅲ) acetylacetonate (0.706 g, 2 mmol) was dissolved in a mixture of benzyl ether (10 mL) and oleylamine (10 mL). The above mixture solution was degassed at 120℃ under argon for 1 h before it was quickly heated to 300℃ and kept at this temperature for another 2 h. The mixture was cooled down to room temperature and then precipitated by the centrifugation with ethanol.

Mixture ligands modified iron oxide nanoparticles (NTF) was prepared by the ligand exchange method due to the specific binding of diphenol hydroxyl group on the iron oxide surface. Briefly, 100 mg iron oxide nanoparticles were dispersed in the mixture of trichloromethane (TCM, 5 mL) and DMF (5 mL). Afterwards, DPA and DPBA-PE with a specific molar ratio and a total mass maintained at 400 mg were added into the ION solution. Then, the mixture was maintained at 50 °C under gentle stirring for 5 h. The NPs were then dispersed in water and dialyzed against 0.01 M hydrochloric acid solution (× 2) and distilled water (× 3). After removing the protection of pinacol, a series of NTF samples (denoted as NTF_n_) with specific ligand ratios (*X*_DPA_: *X*_DPBA_ of 0:100, 16:64, 28:71, 45:55, 66:34, 80:20 and 100:0) was obtained.

**Preparation and characterization of protein loaded nanoclusters:** The bovine serum albumin (BSA) loaded nanoparticles (named as NTF_n_-BSA) were prepared by simply mix at specific mass ratio of 1-5, 1-2, 1-1, 2-1 and 5-1. The suspension was then well-dispersed by ultrasound to obtain uniform nanoclusters. In order to verify the formation of nanoclusters, the particle size and zeta potential was measured by Malvern Mastersizer. Meanwhile, in order to reflect the successful encapsulation of BSA, we monitored the fluorescence quenching effect of BSA-FITC with the NTF_n_ involvement. The preparation of collagenase (CLG)-loaded nanoclusters was similar to above procedure, with the only difference that CLG was selected as the protein drug.

**ATP-triggered size switch and BSA release:** For ATP-triggered size switch and charge reverse study, nanoparticles were reacted with definite concentration of ATP (0, 0.05, 0.1, 0.5 × 10^-3^ M) for 30 min at 37 °C. After that, the particle size and zeta potential were monitored by Malvern Mastersizer. The morphology changes of nanoclusters after ATP incubation was carried out on JEOL JEM-2100f transmission electron microscopy (TEM) at 200 kV.

To investigate the protein release, we monitored the recovery of fluorescence in NTF-BSA-FITC at Ex 488 nm and Em 520 nm. Subsequently, the Fluorescence Resonance Energy Transfer (FRET) technology was performed. BSA was labelled with FITC and acted as the donor of the FRET pair. NTF were labelled with rhodamine B (RhB) and acted as the receptor. The excitation wavelength of the detection was 488 nm and emission wavelength were among 500 nm to 700 nm. The fluorescence spectrum was detected with the increasing ATP (0, 0.05, 0.1, 0.2 and 0.5 × 10^-3^ M) added into NTF-BSA solution.

**Lysosome-specific aggregation of nanotransformers induces cell death:** The bio-transmission electron microscope (bio-TEM) was used to directly observe the lysosomal aggregation of different nanoparticles. Briefly, 4T1 cells were seeded into culture dishes and grew for 24 h. Subsequently, various particles were co-incubated with cells for 4 hours within a weakly acidic environment (pH 6.5) containing a low-concentration of adenosine triphosphate (ATP, 0.1 mM). This experimental condition accurately mimics the pathological state of the tumor microenvironment. After the incubation period, the cell-culture medium was carefully replaced with fresh medium, and the cells were allowed to continue growing until the 24-hour time-point was reached. Subsequently, all samples were collected in a meticulous manner and subjected to observation using a bio-TEM.

To investigate cell uptake and aggregation behaviour of nanoclusters, 1.5×10^4^ cells/well are seeded into confocal dishes and grew for 24 h. Before further treatment, the medium was supplemented with 0.1 mM ATP and maintained at pH 6.5 to mimic tumor microenvironment. Then, the NTF_n_-BSA was added to above cells at a dosage of 10 μM (labelled by Cy3). Subsequently, the nanoclusters-containing medium was removed, and the cells were allowed to incubation for another 20 hours. the samples were meticulously washed three times with phosphate buffered saline (PBS) and fixed using polyformaldehyde. After Staining the cell nuclei with 4',6-diamidino-2-phenylindole (DAPI), the samples were observed under a confocal laser scanning microscope (CLSM, Zeiss, LSM700).

To investigate whether the lysosomal aggregation could increase the retention, the intracellular iron amount after nanoclusters pre-treatment was analysed using ICP-OES. Brieﬂy, 4T1 cells were seeded into 24-well cell culture plates and cultured overnight. Cells were incubated with the different preparations for 4 h before being replaced with fresh medium and continued to grow until 24 h. Then, the cells were collected and lysed for ICP-OES analysis.

**ATP-activated tumor deep penetration:** The *in vitro* ATP activated deep penetration of nanotransformers was evaluated on 3D cell model. Briefly, the 96 well plate was coated with 65 μL 2% agarose gel each well, after cooling, 4T1 cells (2000 cells per well in 200 μL medium) were seeded and cultured for one week. When reached 400 mm, the spheroids were transferred to the confocal dishes and treated by Cy3 labeled nanoclusters (ION-BSA, NTF-BSA and NTF-CLG) for 12 h in the presence or absence of 1 × 10^-4^ M ATP. Afterwards, the spheroids were washed with PBS and fixed by 4% paraformaldehyde. Finally, the images were obtained by CLSM in z-stack with 5 μm intervals. To quantitatively characterize the tumor permeability of the nanotransformers, the above tumor spheres were collected and digested into single cells using trypsin, and the fluorescence signal was analyzed by flow cytometry.

**In vitro lipid peroxidation detection:** The Liperfluo could be oxidized by lipid peroxides (LPO) and emit fluorescence, which was used to evaluate the production of intracellular LPO. 5 × 10^4^ cells per well were seeded at 12-well plates. After incubation overnight, the cells were exposed to PBS, ION, ION-BSA or NTF-BSA (10 μM) for 12 h. Subsequently, the cells were incubated for another 30 min with Liperfluo and collected for FCM analyses. In addition, the LPO level after different treatments was directly observed by fluorescence microscope imaging.

**In vitro ROS generation:** 4T1 cells (5 × 10^4^ per well) were plated on a petri dish and treated with PBS, ION, ION-BSA or NTF-BSA (10 μM) for 12 h. After which, the cells were incubated with 2',7'-Dichlorodihydrofluorescein diacetate (DCFH-DA) to detect intracellular ROS. Finally, DCF fluorescence intensity was immediately observed using fluorescence microscope.

**In vitro GSH determination:** 4T1 cells were cultured on the 6-well plates and co-incubated with PBS, ION, ION-BSA or NTF-BSA (10 μM) for 12 h. After lysed by repeated cycles of freezing and thawing, the treated cells were centrifuged and the intracellular glutathione (GSH) level in the supernatant was measured by Ellman’s assay.

**Cytotoxicity of nanotransformers:** To measure the cytotoxicity of different nanoclusters in tumor cells, 4T1 cells were seeded into 96-well cell culture plates and treatment with different nanoparticles with 1 × 10^-4^ M ATP for 24 h. Then, the cell viability of 4T1 cells were evaluated by 3-(4,5-Dimethylthiazol-2-yl)-2,5-diphenyltetrazoliumbromide (MTT) assay. The same procedure was performed to verify the cytotoxicity of NTF-BSA in four tumor cell lines (including 4T1, B16F10, A549 and HepG2) and four normal cell lines (including L929, L02, 293T and HUVEC).

The tumor cell killing effect of NTF-BSA also confirmed by calcein AM/prodium iodide (PI) double staining. Briefly, the 4T1 tumor cells were seeded into 24-well plates at an appropriate density and plated in a cell-culture incubator for 24 h to allow the cells to adhere and grow. Then, different treatments were performed to the wells. After incubation, the cells were gently washed and stained by calcein-AM/PI working solution. Finally, the stained cells were observed under a fluorescence microscope. Live cells will be stained green by calcein-AM, while dead cells will be stained red by PI. Count the number of live and dead cells in multiple fields of view. The cell-killing rate was calculated to assess tumor cell-killing effect of the drugs, using the follow formula:

Cell-killing rate (%) = (Number of dead cells / Total number of cells) × 100%.

**Detection of Immunogenic Cell Death Biomarkers:** A total of 5 × 10^4^ 4T1 cells were inoculated into the petri dish and subjected to treatment with PBS, ION, ION-BSA or NTF-BSA (10 μM) for 12 h. Subsequently, the cells underwent a standard immunofluorescence assay to assess the surface exposure level of calreticulin (CRT). Afterward, the cells were further stained using Hoechst 33342, and finally observed and imaged under a CLSM. To complement the immunofluorescence results, flow cytometry was further employed for quantitative analysis of CRT surface expression. Briefly, parallel 4T1 cell cultures were gently harvested using non-enzymatic cell dissociation buffer to preserve membrane integrity. The collected cells were washed twice with cold PBS and resuspended. After blocking non-specific binding with 5% normal goat serum for 30 min on ice, cells were incubated with fluorochrome-conjugated anti-CRT primary antibody in the dark at 4°C for 45 min. Then, flow cytometric analysis was performed to quantitatively detect the surface expression of CRT in 4T1 cells after different treatments.

To assess the release of high mobility group box-1 protein (HMGB1), 100 μL of supernatant was collected from each well containing cells treated with different formulations at a concentration of 10 μM. This supernatant was then analysed using an enzyme-linked immunosorbent assay (ELISA) kit in strict accordance with the manufacturer’s recommended protocol. In addition, a chemiluminescence-based ATP Determination Kit was employed to carry out the measurement of ATP.

**In vitro DC stimulation**

The assessment of DC maturation induced by immunogenic cell death (ICD) was conducted using a 24-well transwell system equipped with 0.4 μm polycarbonate porous membranes. Specifically, 4T1 cells were first seeded into the upper chambers of the transwells and permitted to adhere for a 12-hour period. Afterwards, the cells were treated with PBS, ION, ION-BSA or NTF-BSA at concentration of 10 μM. Then, the upper chambers containing the treated 4T1 cells were transferred to new transwell plates and co-incubated with DCs that had been pre-plated in the bottom chambers. After an additional 12-hour co-incubation period, the DCs were harvested and subjected to co-staining with anti-CD80-APC, and anti-CD86-FITC antibodies. Finally, the stained DCs were analysed using flow cytometry to evaluate DC maturation status.

**In vitro macrophage polarization assay:** RAW264.7 cells were seeded in the lower chamber and co-incubated with the upper chambers containing the 4T1 cells. The different formulations (10 μM) were added to the upper chamber. After 24 hours of incubation, total RNA was extracted from the treated RAW264.7 cells. RT-PCR was then performed to detect the expression levels of M1-related makers (CD86 and iNOS) and M2-ralated genes (CD206 and Arg-1), according to the manufacturer’s recommended procedure.

**Animals:** Female BALB/c mice (18-22 g) were maintained under appropriate conditions. All animal experiments were approved by the Animal Ethics Committee of China Pharmaceutical University (2025-2-085).

**In vivo biosafety evaluation:** Healthy mice were randomly divided into different groups and intravenously administered with saline, ION, ION-BSA, NTF-BSA and NTF-CLG (10 mg/kg), respectively. After continuous treatment, blood samples were collected for routine blood examination and serum biochemistry analysis to evaluate hematological status, as well as liver and renal function. In addition, body weight changes were monitored throughout the experiment to evaluate the systemic biosafety and biocompatibility of the nanotransformers in vivo.

**In vivo antitumor efficacy of lysosomal aggregation induced cell death:** To further assess the in vivo antitumor efficacy of NTF-CLG, 4T1 xenograft model was constructed. The tumor bearing mice were randomly divided into four groups (n = 5) and intravenous (i.v.) injected with saline, ION, ION-BSA, NTF-BSA and NTF-CLG (10 mg/kg), respectively. The administration was performed every three days for a total of three times. The tumor growth was monitored by measured their volumes every two days. Tumor volumes were calculated according to the following formula:

Tumor volume (V, mm^3^) = 0.5 × length × width^2^

After intravenous administration of different treatments to 4T1 tumor-bearing mice, tumors were collected on day 14 and stained with Masson’s trichrome. The survival time of mice in each group was monitored until the tumor volume reached 1500 mm³ or until 60 days post administration.

The immune activation effect was assessed following three administrations. On day 14, the 4T1 tumor-bearing mice were sacrificed, and their tumors and tumor-draining lymph node (TDLN) were collected. These harvested tumor tissues were then processed through mechanical disruption, digested and filtered to obtain single-cell suspensions. Subsequently, the cells were subjected to counting, followed by staining with a panel of specific antibodies in strict accordance with the protocols provided by the manufacturer. For the assessment of DC maturation, cells isolated from TDLN were stained with a panel of conjugated antibodies, including anti-CD11c-PE, anti-MHC II-PE/Cy7, anti-CD80-APC, and anti-CD86-FITC antibodies. Following the staining procedure, the cells were analyzed using flow cytometry to determine DC maturation status.

The standard percoll experimental procedure was used to isolate lymphocytes from the single-cell suspension of the tumors. Then, the lymphocytes were stained with anti-CD45-perCP/Cy5.5, anti-CD3-FITC, anti-CD4-APC/Cy7, anti-CD8-PE-Cy7 and further determined via the flow cytometry. In addition, the ex vivo fluorescence staining was performed to evaluated the immune cells in the tumor sections. Briefly, tumors were excised after treatment, and collected to prepare tumor slices. Then these tumor sections were sequentially stained with anti-CD8, anti-CD4 antibodies and DAPI with a standard procedure. Finally, the tumor slices were imaged using fluorescence microscope.

In parallel, tumor derived cells were stained with a comprehensive panel of antibodies including anti-CD45-FITC, anti-CD11b-Percp-Cy5.5, anti-F4/80-PE, anti-CD86-BV421, anti-CD206-AF467 to assess the macrophage polarization status within the tumor microenvironment.

To investigate the expression levels of tumor associated cytokines, tumor necrosis factor-α (TNF-α) and interleukin (IL-6) in the supernatant of tumor homogenates were examined using enzyme-linked immunosorbent assay (ELISA) kits.

**Statistical analysis:** All statistical analyses were conducted on GraphPad Prism version 10 software. Data from the experiments were performed for three times or over three times and the results are presented as the mean ± standard deviation (SD) unless otherwise noted. A two-tailed student’s t-test was performed for statistical analysis of the difference between the two groups. *P value < 0.05* was considered statistically significant between the data sets, where all significant values were indicated as follows: **p< 0.05*, ***p< 0.01*, ****p< 0.001*, *****p< 0.0001*.

**Figures**

**Figure S1.** Transmission electron microscopy image of the iron oxide nanoparticles.

**Figure S2.** TEM-EDS elemental mapping of the iron oxide nanoparticles.

**Figure S3.** XRD spectra of the iron oxide nanoparticles. The XRD pattern of the as-prepared ION powder shows diffraction peaks corresponding to standard XRD data of magnetite (Fe_3_O_4_, JCPDS No. 19-0629).

**Figure S4.** Mass spectrometry of pinasterol ester protected DPBA.

**Figure S5.** Characterization of the pinasterol ester protected DPBA by ^1^H NMR.

**Figure S6.** The particle size of NTF-BSA nanoclusters with *X*_DPA_:*X*_DPBA_ of 45:55 at different time points under different conditions. (n = 3, mean ± SD).

**Figure S7.** FTIR spectra of BSA, ION, NTF and NTF-BSA.

**Figure S8.** (a) Thermogravimetric analysis (TGA) curves and (b) derivative thermogravimetry (DTG) curves of BSA, ION, NTF, and NTF-BSA nanoclusters under a nitrogen atmosphere.

**Figure S9.** DLS analysis of NTF-BSA in different conditions. (a) The particle size of NTF-BSA following incubation in solutions at different pH values supplemented with 0.1 mM ATP. (b) The particle size of the NTF-BSA in solutions with different pH values in the absence of ATP.

**Figure S10.** Particle sizes of NTF-BSA and NTF-CLG nanoclusters under three distinct pathological microenvironments.

**Figure S11.** FACS analysis of 4T1 MCSs cells treated with nanoclusters. Cells in the squares were regarded as Cy3-positive cells.

**Figure S12.** Cell viability of DCs treated with NTF-BSA at optimal transduction conditions for 24 h (n = 3, mean ± SD).

**Figure S13.** Quantitative analysis of the M1-related makers (a) CD86 and (b) iNOS in RAW264.7 cells after incubated with different formulations. (n = 4, mean ± SD).

**Figure S14.** Quantitative measurement of the M2-related makers (a) CD206 and (b) Arg-1 in RAW264.7 cells by polymerase chain reaction. (n = 4, mean ± SD).

**Figure S15.** Representative flow cytometric analysis of mature DCs in lymph nodes following various treatments.

**Figure S16.** Flow cytometry analysis of MHC II^+^ DCs with different treatments.

**Figure S17.** In vivo biosafety evaluation. (a-g) Hematology and (h−l) blood biochemistry analysis of healthy mice after being treated with different formulations (n = 4, mean ± SD).

**Figure S18.** Histological assessment of the major organs (heart, liver, spleen, lung, and kidney) by H&E staining after treatment with different formulations. Scale bar, 500 μm.
